# Website for families of non-breastfed children: development and validation of content and interface

**DOI:** 10.1590/0034-7167-2023-0490

**Published:** 2024-06-17

**Authors:** Marília Alessandra Bick, Cristiane Cardoso de Paula

**Affiliations:** I Universidade Federal de Santa Maria. Santa Maria, Rio Grande do Sul, Brazil

**Keywords:** Food Security, Infant Health, Family, Translational Science, Biomedical, Educational Technology, Seguridad Alimentaria, Salud del Lactante, Familia, Ciencia Traslacional Biomédica, Tecnología Educacional

## Abstract

**Objectives:**

to develop and validate the content and interface of a guidance website to support families in promoting Food and Nutrition Security for children under six months who are not breastfed.

**Methods:**

methodological study, Knowledge Translation, in two stages of creation: 1) content and validation on the criterion of accuracy in a panel of experts; 2) interface and validation on the criteria of content, language, illustrations, layout, motivation, culture and applicability.

**Results:**

the “Milky Way” website is freely available: https://www.ufsm.br/pet/ciencia-da-computacao/alimentacao-lactea. The content was structured in a decision tree made up of types of milk: milk formula, whole cow’s milk and powdered milk; and utensils: bottle, cup and measuring spoon. There were 46 illustrations to elucidate the content, facilitate understanding and engage the target population. The Content Validity Index was 0.91.

**Conclusions:**

the website is a validated technology with evidence-based written and pictorial content translated for use with families.

## RESUMEN

**Objetivos:**

desarrollar y validar el contenido y la interfaz de sitio web orientador para apoyar a familias en la promoción de Seguridad Alimentaria y Nutricional de niños menores de seis meses no amamantadas.

**Métodos:**

estudio metodológico, de Traducción del Conocimiento, en dos etapas de creación: 1) contenido y validación según el criterio de precisión por panel de expertos; 2) interfaz y validación según los criterios contenido, lenguaje, ilustraciones, maquetación, motivación, cultura y aplicabilidad.

**Resultados:**

el sitio “Vía Láctea” es de libre acceso: https://www.ufsm.br/pet/ciencia-da-computacao/alimentacao-lactea. El contenido se estructuró en un árbol de decisión compuesto por tipos de leche: fórmula láctea, leche entera de vaca y leche en polvo; y utensilios: biberón, taza y cuchara dosificadora. Hay 46 ilustraciones para elucidar el contenido, facilitar la comprensión y atraer a la población objeto. El índice de validez del contenido fue de 0,91.

**Conclusiones:**

el sitio web es una tecnología validada con contenidos escritos y visuales basados en pruebas y traducidos para su uso con las familias.

## INTRODUCTION

There are few conditions in which it is recommended that women do not breastfeed, such as mothers infected with HIV, HTLV 1 and 2 or taking medication that is incompatible with breastfeeding, such as cancer treatment. Mothers who are regular users of alcohol or illicit drugs should not breastfeed while using these substances. In the case of mothers with disorders of consciousness or severe behavior, the case should be assessed separately. In any of these situations, feeding the non-breastfed child must necessarily be acceptable, feasible, accessible, sustainable and safe^(1,2)^. The concept of food and nutritional security (FNS) incorporates aspects related to the availability, production, marketing and access to food in the food dimension; and aspects related to the selection, preparation and consumption of food, its relationship with health and the use of nutrients in the nutritional dimension^(3)^.

For this to happen, it is essential that the family has drinking water, basic sanitation at home and the financial means to provide infant formula and food in sufficient quantity for the child to grow properly. It also requires the knowledge and skills to prepare them in adequate quantity and quality and to offer them in an age-appropriate manner. It requires regular access to health services to monitor children’s growth and development^(4,5)^.

The exposure of these children to inadequate nutrition due to the absence of breastfeeding increases their predisposition to infant morbidity and mortality^(6,8)^. For this reason, they need to be guaranteed the right to adequate and healthy food, and for this to be possible, family members need to receive guidance and information based on scientific evidence, in a timely manner, in an accessible way and without conflict of interest, so that they can adopt safe feeding practices^(5,9)^.

However, these family members receive insufficient support and guidance. For this reason, the families’ main source of information is the infant formula can itself, which contains specific information on how to prepare the formula using a bottle and how to dilute the formula according to the infant’s age. Mothers, who are primarily responsible for feeding the child, are dissatisfied with the amount and variety of information available to help with infant formula feeding^(5)^.

Health professionals are key to supporting the families of children under six months of age who are not breastfed, informing them about the types of milk and infant formula available, the types of utensils available to offer the milk, the practices for sanitizing the utensils, how to prepare it, the quantities and the care needed to ensure that the child receives the nutrients necessary for proper growth and development^(5)^. The nurse and pediatrician in primary health care services should guide the families of these children according to the recommendations on feeding children in the first years of life, which support professionals in the development of individual and collective food and nutrition education actions in the Unified Health System^(2)^. Community health workers should monitor the demands of these families and children during home visits.

The daily dietary practices of infants change with age and family members are not always able to access health professionals to clarify their day-to-day doubts, given that the schedule of childcare appointments is monthly in the first six months of life. Educational technologies are an alternative that can help strengthen guidelines, but there is a lack of these up-to-date tools based on scientific evidence about the feeding of non-breastfed children. For this reason, parents and/or other people involved in the daily feeding practices of infants often receive conflicting information from inadequate sources^(4,9)^.

In particular, digital technologies meet the growing information needs of the population, who are trying to manage their health using online resources. Various digital tools are capable of enabling interaction between the population and the health system, making them a valuable ally. Thus, the literature describes the use of the web as a successful strategy for accessing health advice^(10)^.

However, there is a need to provide a reliable source of information based on scientific evidence and to translate the guidelines to promote nutrition and healthy child development. Considering the wide availability of existing scientific evidence and the difficulty the population has in accessing this information, presenting it in accessible language and adjusted to the guidelines of health professionals makes it possible to qualify health care and bring research closer to clinical practice.

## OBJECTIVES

To Develop and validate the content and interface of a website to support families in promoting Food and Nutrition Security for children under six months who are not breastfed.

## METHODS

### Ethical aspects

This research followed Resolutions 466/2012, 510/2016 and 674/2022, with a matrix project approved by the Research Ethics Committee. The privacy and confidentiality of personal information was guaranteed by the Data Confidentiality Agreement through the sending of individual messages and emails, the application of the free and informed consent form electronically, and the questionnaire could be answered with the participant’s electronic acceptance, clarification of the possibility of abstaining from the research at any time without any moral or legal harm, and storage of the database in a secure way that makes it impossible to identify the participant.

### Study design, period and location

**Figure 1:**
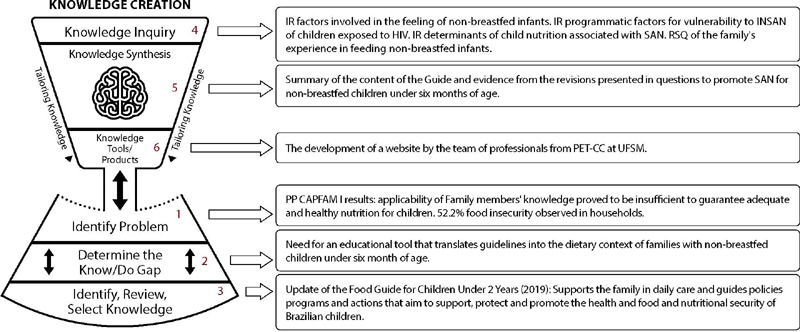
Application of the phases of the creation cycle in the Knowledge Translation Project, Santa Maria, Rio Grande do Sul, Brazil, 2022

The design of this research is part of a matrix project on knowledge translation (CT)^(11)^ entitled “Translation of knowledge in the production of educational technologies to promote care for children at risk of exposure to HIV” (CAPFAM III), developed by the Research Group on Health Care for People, Families and Society (GP-PEFAS). In the sub-project that gave rise to this article, the cycle for creating the CT Model was developed (Figure 1).

*IR - Integrative Review; INSAN - Food and Nutritional Insecurity; SAN - Food and Nutritional Security; RSQ - Qualitative Systematic Review; PET-CC - Tutorial Education Program of the Course in Computer Science; PP - Research Project; CAPFAM - Evaluation of the capacity to care for children exposed to HIV.*

*Source: Graham, Logan, Hanson, Strauss, Tetroe, Caswell, Robinson^(12)^ (translated by Vieira, Gastaldo, Harrison^(13)^).*

**Figure 2:**
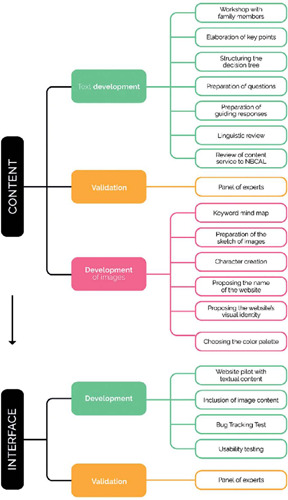
Methodological flowchart of the Knowledge Translation Project, Santa Maria, Rio Grande do Sul, Brazil, 2022

The creation period for this technology took a total of 40 months and included: 1) the content creation stage (text development, validation and image development) and 2) the interface creation stage (website development and validation) (Figure 2).

Content development (knowledge synthesis phase) began in December 2019, following the update of the Food Guide for Brazilian children under 2 years of age^(2)^. The text and image content was developed over 24 months and this phase was completed in December 2021 with the validation of this content by a panel of experts. Development of the interface (knowledge product phase) began in April 2022 and lasted 12 months, with validation of the interface by experts in November 2022. In May 2023, the website obtained a registration number and was unveiled at a launch event.

The study site is a Brazilian public and federal higher education institution in the countryside of the state, whose mission is to build and disseminate knowledge, committed to training people capable of innovating and contributing to the development of society in a sustainable manner. This mission, which is consistent with the Institutional Development Plan, which includes the topic of innovation and technology transfer in the guidelines for teaching, research and extension policies, provided institutional support for the development of this technology and inter-sectoral and multidisciplinary coordination for the composition of the CT project team.

### Population or sample; inclusion and exclusion criteria

To validate the content, a panel of experts was selected using the snowball technique. This is a non-probabilistic sample, and this technique was chosen due to the need for evaluation by professionals with knowledge and experience in the subject of feeding and in the population under the age of six months.

The composition of the panel of experts with representatives of health professionals, teachers, municipal, state and federal managers with experience in the subject was a challenge that was taken up by the TC Model, which provides for this engagement of decision-makers. This challenge was achieved through the contacts established during the development of the projects that make up the GP’s food and nutrition security nucleus (NUSAN), including the numerous opportunities on which the CAPFAM matrix project was presented at events, including awards, which gave it national and international visibility.

Initially, the invitation was sent to the general technical coordinator of the Guide^(2)^, The Ministry of Health’s General Coordination for HIV Surveillance, the Regional Health Coordination and the Municipal Health Department. The invitation was sent by e-mail and upon receiving it the professional could nominate other specialist(s) to make up the panel, to whom the invitation was also sent. Twenty-five health professionals from different locations and institutions across the country were invited. Thirteen experts took part in the panel, all of whom were linked to the following institutions: General Coordination of Food and Nutrition (CGAN) of the Ministry of Health; International Network in Defense of the Right to Breastfeed (IBFAN); United Nations Children’s Fund (UNICEF); National Council for Food and Nutrition Security (CONSEA); Pan American Health Organization (PAHO); HIV TV Response Center of the Ministry of Health; Food and Nutrition Policy of the state of Rio Grande do Sul; 4th Regional Health Coordination; Municipal Health Secretariat; Municipal HIV/AIDS Policy; and Pediatrics of the University Hospital of Santa Maria.

To validate the website interface, participants were recruited using the Snowball technique. Initially, the invitation was sent to the 13 experts who took part in the content validation, and those who agreed to take part in this stage of validating the interface indicated other experts on the subject. A total of 77 invitations were sent out, of which 28 experts agreed to take part.

### Study protocol

This study was developed in two stages: 1) creation of the content (text, language, illustrations and visual identity) based on scientific evidence; and 2) development of the interface (layout and operability), both phases validated by experts.

The first stage began with a workshop at the higher education institution with representatives of family members of children exposed to HIV because they do not receive breast milk. The family members were invited to take part through an extension project being developed by the research group in the specialized health service at the University Hospital, and participation was voluntary. In this workshop, the content was presented in the form of questions and guidelines. The aim was to check that the website proposal met the needs of the target population and was consistent with their demands and the local context. The participants talked about the experience of feeding children, their difficulties and the reality of their daily lives, which helped to develop the textual and imagery content.

To develop the textual content, key points were selected from the scientific evidence and the Guide. Subsequently, the decision tree was structured in which the key points were grouped by type of milk. The content of this tree was made up of questions, with the option of dichotomous answers (yes or no). In order to write the questions, the families’ experiences of preparing and offering milk-based meals, which were accessed during the workshop with the target audience, were taken into account.

A panel of experts was assembled for linguistic revision. This stage was carried out virtually, through two meetings with a specialist in the field of Literature and another specialist in the field of Advertising. After the linguistic review, a panel was held with two experts from the Monitoring Group of the Brazilian Standard for the Commercialization of Food for Infants and Young Children, Nipples, Pacifiers and Bottles (NBCAL). In this panel, adjustments were made to the text and images in the material in order to meet the recommendations of the Standard.

The textual content was validated by a panel of experts in infant feeding. The invitation was sent by e-mail, which contained a presentation of the research project’s objectives, an indication of the possible dates for the panel and a link to an electronic agenda (Doodle) for selecting the best shift and date. After confirming acceptance, the file was sent for prior appraisal.

In the panel discussion, the textual content was reviewed by experts for evidence of content validity, paying attention to the accuracy criterion^(14)^ of the questions and guidelines. Participants sent in their suggestions for adjustments, which were compiled (and highlighted in red) in a single file by the panel mediator. This was shared on the meeting screen, which took place over two days, totaling seven hours. The mediator read out the content of each question. During the process, they had the opportunity to suggest deletions, additions and changes to the text and image content. When an expert indicated a change, the text was adjusted and, after the panel reached a consensus, the next question in the decision tree was validated.

An illustrator was hired and virtual meetings were held with him. Communication was maintained via messaging application to optimize communication. A script was drawn up according to the validated content of the questions and guidelines, which detailed the characteristics required for each image. Initially, the illustrator presented a mind map with keywords that helped him construct the illustrations and sketches of the images of the types of milk and the utensils for feeding the child. Then the characters were created. The images were then presented and the research team asked for adjustments. Adobe Photoshop was used with a graphics tablet.

The research team and the illustrator worked together to choose the name of the website, researching terms that referred to the content, without prejudice to compliance with NBCAL. The illustrator then presented the technology’s visual identity. Tests were then carried out to choose the main colors for the palette. A survey of colors for representing skin color was also carried out, being as comprehensive as possible, aiming for diversity. Once the color palette was defined, the visual identity and illustrations were finalized with the addition of colors, lights and shadows.

The second stage began with a partnership with a team of computer scientists linked to the Computer Science Tutorial Education Program (PET-CC). The virtual meetings made it possible to present the proposal, clarify doubts and make adjustments. Communication was maintained by e-mail and messaging application. A decision tree script was drawn up with the questions and the content of the yes answer and the guidance for the no answer, with their respective images. To develop the interface, the PET-CC team developed a pilot website with textual content, which was accessed by the research team who drew up a report on the necessary adjustments. The visual identity and illustrations were then added. The WordPress platform was used to create and maintain the website. The Elementor tool was used for detailed and personalized editing of the pages. This software was chosen because of the need to host the university’s institutional website, which uses the platform in question.

The research team carried out a problem-tracing test on the website, going through the decision tree and accessing the links, which generated a second report on the adjustments needed. Finally, the research team carried out the first usability test, with the website being accessed via cell phone, tablet (Android and IOS) and computer, seeking to identify the need for font and color adjustments and system corrections. Any non-conformities identified were reported. Some of the functionalities desired by the team were not possible due to a limitation of the institutional platform, such as the impossibility of reading the entire page when accessed via a smartphone with an Android operating system.

The interface was validated by a panel of experts. The invitation was sent by e-mail, in which the objective of the research, the method used for validation and the access link to Google Forms were presented. To ensure the participation of a greater number of experts, reminder emails about the survey were sent every five days to participants who had not responded. Contact was also made via the WhatsApp application. Validation was carried out using an online form, using the Google Forms platform, linked to the researcher’s institutional e-mail address.

The first part of the form consisted of characterizing the experts, with seven questions. Next, the participant was invited to access and browse the website and then answer the validation form, which has already been applied to educational technology for families^(15)^. The validation criteria were: a) content - with five items; b) language - with three items; c) illustrations - with five items; d) layout - with seven items; e) motivation - with three items; f) culture - with two items; g) applicability - with one item. A Likert-type scale was used for each question, with the options: 1 - Inadequate; 2 - Partially adequate; 3 - Adequate; 4 - Totally adequate. At the end of each block of questions, a space was provided for the experts to make suggestions and opinions for improving the technology.

### Analysis of results and statistics

The answers were exported from the Google Forms platform, in Microsoft Excel file format, to the SPSS v. 20.0 analysis program. Categorical variables were described by frequencies and percentages. The normality of the quantitative variables was assessed using the Shapiro Wilk test. Quantitative variables with a normal distribution were described by the mean and standard deviation (age) and those with an asymmetric distribution by the median and interquartile range (length of service and training).

The Content Validity Index (CVI) was calculated for each item, category and overall. The averages were used to calculate the CVI for the categories: content, language, illustration, layout, culture and applicability. The CVI measures the percentage of agreement between experts on certain aspects of the instrument and its items. The CVI can be calculated for each item and for the total scale. To calculate it, the scale needs to have four points (1 - Inadequate; 2 - Partially adequate; 3 - Adequate; 4 - Fully adequate), answers 3 and 4 are added together and the result of this sum is divided by the total number of answers. An acceptable CVI for each of the items should be at least 0.78 and for the overall scale 0.80. Lower values indicate the need to revise or reject the items^(16)^.

The instrument’s Cronbach’s alpha was calculated. The minimum acceptable value for reliability is 0.70^(17)^. *Cronbach’s alpha was high at 0.95. This result indicates that, although the instrument used was not designed for this topic or for this specific population, it was suitable for data collection, showing internal consistency of the items*.

## RESULTS

The website’s visual identity is “Milky Way: the path to food and nutritional security for children under six months who are not breastfed”. This choice was based on the identification that the Milky Way Galaxy was so named by the Greeks because of its whitish appearance and was seen as a path of milk. Therefore, the name “Milky Way” refers both to the route taken by the family to feed the child who is not breastfed, and to the way in which the child is fed. And the term dairy is defined by the dictionary as “containing milk, composed of milk”.

The main colors chosen were green and pink, which are complementary, contrast and highlight the elements in the images. Colors similar to pink were sought and, to broaden the possibilities, brown and yellow were included. The tones vary between vibrant, saturated colors and pastels.

The website’s home screen contains a brief presentation of the purpose of the technology, the target audience, the justification for the family’s need to obtain FNS guidance for non-breastfed children under six months and what FNS means. There is a link to the Food Guide for Brazilian children under 2, the main source of content for this technology. It also reinforces the need for the family to seek out a health professional and provides links to the Child Health Handbook.

**Figure 3:**
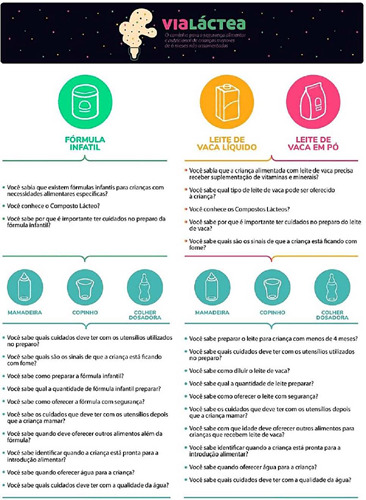
Visual identity and website decision tree, Santa Maria, Rio Grande do Sul, Brazil, 2022

On the second screen is the decision tree, where the family member can choose the path to follow on the website, according to their need for guidance. The textual content was structured in questions and answers, with choices by type of milk: milk formula with 4 questions, whole cow’s milk and powdered milk with 6 questions each; and utensils: bottle, cup and measuring spoon, with 10 questions each (Figure 3).

**Figure 4:**
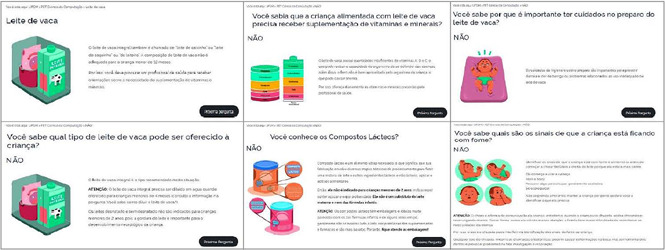
Mosaic of screenshots of the website on the option of a glass utensil for whole cow’s milk, Santa Maria, Rio Grande do Sul, Brazil, 2022

There are 46 illustrations to elucidate the content, facilitate understanding and engage the target population. Icons were created to identify the three types of milk and the three types of utensils used to feed the child. The characters are: a child under six months old, a health professional, a mother, a father and a school-age sibling. Images were also created of materials that families use in their daily lives to prepare milk and to clean utensils. The illustrations were linked to the textual content, as can be seen in the example of some guidance screens in Figure 4.

At the bottom of each screen, you can click on the summary option, which redirects you to the decision tree screen and allows you to select the type of milk and utensil used to feed the child again. In this way, the journey can be carried out as many times as necessary.

The final screen of the website includes credits for authorship, illustration, website programming, textual revision, compliance with NBCAL and experts from the validation panel. In addition to the work registration seal, information on the innovation project award and the funding agencies.

The website obtained a Content Validity Index of 0.91. The content, language, illustration, layout, motivation, culture and applicability criteria all obtained CVIs within the previously established parameters (Table 1). Although the website had an adequate overall CVI, the changes proposed by the experts to the images and text were taken into account for the final version.

**Table 1:** Distribution of the Content Validation Index in the panel of experts, Santa Maria, Rio Grande do Sul, Brazil, 2022

CRITERIA/Items	CVI
CONTENTS	0.85
A1. The content is scientifically correct	0.86
A2. The content is appropriate for the target audience	0.75
A3. The content is sufficient to meet the needs of the target audience	0.82
A4. The sequence of the text is logical	0.89
A5. The presentation of the content is conducive to learning the topic	0.93
LANGUAGE	0.89
B1. The writing style is compatible with the target audience	0.96
B2. The writing is attractive	0.89
B3. The language of the text is clear and objective	0.82
ILLUSTRATION	0.93
C1. The illustrations are relevant and elucidate the content	0.86
C2. The illustrations are clear and easy to understand	0.89
C3. The illustrations have graphic quality	0.96
C4. The number of illustrations is appropriate for the content of the material	0.93
C5. The presence of each figure is relevant	1.00
LAYOUT	0.94
D1. The font used makes it easy to read	0.96
D2. The colors applied to the text are relevant and make it easier to read	1.00
D3. The visual composition is attractive and well organized	0.93
D4. The format of the educational material is appropriate	0.90
D5. The layout of the text is appropriate	0.89
D6. The font size of the headings, subheadings and text is appropriate	0.96
D7. The size of the technology is appropriate	0.93
MOTIVATION	0.94
E1. The content is motivating and encourages further reading	0.93
E2. The content aroused the reader's interest	0.96
E3. The content can answer questions, clarify and educate the family member	0.93
CULTURE	0.87
F1. The text is compatible with the audience, catering for different profiles	0.82
F2. The technology is suitable for use as a family support resource	0.93
APPLICABILITY	0.93
G1. The technology has practical applicability	0.93
Total	0.91

*CVI – Content Validation Index.*

A total of 28 experts responded to the validation, of whom 27 (96.4%) were female and 1 (3.6%) male. The average age of the participants was 43.4 years. With regard to the area of training, 15 (53.6%) were from nursing, 9 (32.1%) from nutrition and 4 (14.3%) from medicine. As for their area of activity, 23 (82.1%) worked in teaching, 20 (71.4%) in research and 11 (39.3%) in care. It should be noted that in this item, the specialists had the possibility of ticking more than one option. The median length of training for the participants was 16 years, ranging from a minimum of 10 to a maximum of 24 years. The median time since the participants’ last degree was 7.5 years, ranging from a minimum of 4.0 years to a maximum of 10.7 years.

With the textual content, images and interface validated, the website obtained the registration number 7121883. It is available on the freely accessible institutional domain: https://www.ufsm.br/pet/ciencia-da-computacao/alimentacao-lactea

## DISCUSSION

The website was validated for use with families to promote FNS in children under six months who are not breastfed. Similar global CVI parameters were found in the validation of other Brazilian educational technologies, ranging from 0.84-0.99, which points to the alignment of the result obtained by the panel of experts with the indices of previous methodological studies of educational technologies on the subject^(18)^ and the target population^(19)^.

The validation of the content criterion indicates that the experts considered the text to be coherent with current scientific evidence, with an appropriate logical sequence, and that it favors learning on the subject. In other methodological surveys carried out in the country, the experts also considered the content of the technologies to be adequate^(20,21)^. The item that scored lower than the cut-off point was the suitability of the content for the target audience, without invalidating the validation of the criterion. The main suggestion made by the experts on this item refers to the suitability of the text containing technical terms. This result indicates the relevance of the next stage of the matrix project, which is to validate the technology with the target audience^(22)^, so that adjustments can be made to the local context, as recommended in the TC Model^(11)^.

Validation of the language criterion indicated that the writing style is compatible with the target audience, that the writing is attractive, clear and objective. In educational technology, language is essential for the message to be understood correctly and to be applicable in people’s daily lives. For this reason, it must be appropriate to the educational and cultural level of the target population, breaking down the barrier between scientific knowledge and tacit knowledge^(21,23)^. It should be emphasized that the use of educational technology also depends on attractive language, which draws the attention of the user of the knowledge translated into the tool. Otherwise, the individual may seek information and guidance from other sources, which are not always appropriate to their needs and supported by scientific evidence^(21,22,24)^. Access to this type of information can increase the risk of food insecurity among children who are not breastfed. The language should be objective to ensure that the family member has time to consult the information and clarify doubts, taking into account the practicality that a caregiver of a child under six months needs^(4,5)^.

Validation of the illustration criterion showed that the website’s illustrations were considered to be of good quality, relevant, clear, elucidating the content and facilitating understanding of the text. The use of illustrations in educational technologies arouses the user’s interest and helps them understand the content. Similar results were found in the development of educational resources for breastfeeding^(18)^ and to prevent overweight^(21)^.

The validation of the layout criterion indicates that the experts considered the website to have an attractive visual composition, with letters and colors that make it easy to read the text, with an appropriate arrangement of letter sizes and text sizes. Two types of font are used on the website: Rawline and Sans Serif, the page background is white and some details found on the page are related to the website being hosted on a UFSM institutional domain. Other Brazilian studies that have built and validated m-Health educational technologies on a wide range of topics reinforce the importance of presenting an attractive and relatively simple layout that allows users to access it regardless of the speed of their mobile network or the storage space on their device^(22,25)^.

For the motivation criterion, the experts consider that the content encourages reading, arouses interest and has the potential to clarify family members’ doubts. Similar results were found in Brazilian studies that validated educational technologies for family members of children. In the validation of the mobile device on breastfeeding for the population of family members of newborns, the motivation criterion obtained a CVI of 0.95^(22)^. The validation of a package of text messages and images to promote breastfeeding obtained a CVI of 0.86 in terms of motivation^(21)^.

In relation to the culture criterion, the experts validated the text as compatible with the population, indicating its use as a resource to support the family in promoting the FNS of children under six months who are not breastfed. Validation studies of educational technologies also showed CVI scores above 0.80 for the culture criterion, which indicates their suitability for use with the Brazilian population^(22,26)^. The experts who took part in the validation of the Milky Way website come from different regions of the country. The validation of the last criterion, applicability in practice, points to the website’s potential for use. The experts indicated the possibility of using the technology in the practice of nursing consultations^(23)^.

The validation of technologies based on scientific evidence by specialists enhances their use, since they are the professionals who provide health care to individuals and who can effectively mediate the use of the educational resource^(21,23)^. Thus, in order to make the technology more complete, suitable for the target population and with greater scientific rigor for use in health education, the suggestions indicated by the experts were taken into account, which has also been identified in other studies^(20,23)^. These suggestions have helped to improve the technology in terms of text and image content, both in line with scientific evidence and in terms of translating knowledge to the target population of family members of children under six months who are not breastfed and to the local context of the Brazilian Unified Health System.

The quality of a care-educational technology^(27)^ is based on the use of scientific evidence and validation by specialists so that it is in line with practice^(28)^. Other Brazilian studies that have developed and validated educational technologies have also used the CVI, indicating the convergence of the method used to qualify the production of these educational resources^(20,24,29)^. In order to incorporate technologies into different scenarios, it is essential that they are validated and that the researcher commits to keeping them under constant evaluation and monitoring^(12,13)^. The development of technologies by multi-professional teams broadens the range of scientific and practical knowledge to improve products^(20,24,30)^. The inclusion of professionals working in the areas of teaching, research and/or care can guide the use of technology as a tool for promoting health^(20,21)^.

Considering the TC model, in order to meet the commitment to disseminate the knowledge tool, the launch of the educational technology was developed through an extension action by the GP called the Seminar for Integrating Research with Practice (SIPP), which is developed at the end of matrix projects to share the results with the community. In this edition, the SIPP took the form of an open class entitled “Food safety for children under six months old”, which was publicized through the social networks of the GP, the Health Sciences Center and the University’s Dean of Extension. It was attended by undergraduate and residency health students, primary care and hospital professionals, teachers and managers. The event was covered by TV Campus and reported on https://www.youtube.com/watch?v=73FRgE0RPPU.

**Figure 5:**
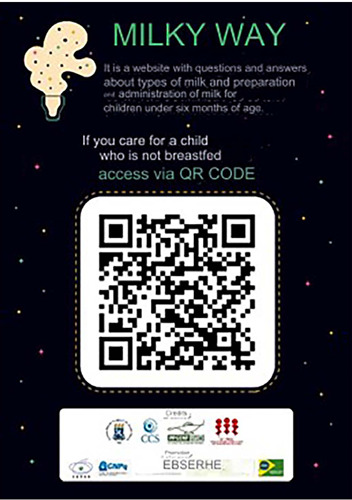
Artwork for the website promotion kit, Santa Maria, Rio Grande do Sul, Brazil, 2022

In accordance with the TCA Model, which advocates stakeholder engagement in order to maintain the use of the knowledge product, the SIPP saw the participation of managers from the University’s teaching, research and extension sectors, the University Hospital’s maternal and child area, the Municipal Health Department and the 4th Regional Health Coordination (CRS). At the SIPP, the website was symbolically handed over to managers by means of a card with a QR CODE for access (Figure 5). A video was also produced with the website production team and the managers who supported it as a tool that could be used in a didactic way as an illustration of the TC engagement process. This video can be accessed at https://youtu.be/d9VJfRG1JkU?si=kAZavT9wR_Y5SyZp.

This same artwork is printed in poster format and made available for dissemination at the services. A website dissemination kit was sent to each municipality in the 4thCRS. The number of cards and posters was calculated according to the number of health units accompanied by a letter from the GP, which also contains the link to the short video promoting the website, available at https://www.instagram.com/reel/CurPY2XuRb/?igsh=NzBmMjdhZWRiYQ. These dissemination products are the result of this CT project and express the commitment to continue disseminating them, as there is a demand for investment to maintain the use of this technology by families of children under six months who are not breastfed.

### Study limitations

The limitation of this study was the result of isolation due to the COVID-19 pandemic, which made it difficult to include the target population in the entire process of developing the website’s content and interface, as recommended by the CT Model to engage the audience of the knowledge product. We therefore suggest that this technology needs to be evaluated by the family members themselves, a stage that is planned in the CAPFAM III matrix project’s implementation schedule.

### Contributions to the field

The contribution is of an innovative nature for the CT Model, with the production of a technology for food and nutrition education according to the needs of the vulnerable group of children under six months of age who are not breastfed. The website can be used by family members in their daily practice of feeding their child. Professionals can mediate its use as a tool for reviewing guidelines and clarifying family members’ doubts. Professionals can be trained to use the website, including updating their practice based on current scientific evidence.

## CONCLUSIONS

The website has been validated in terms of content, language, illustrations, layout, motivation, culture and applicability. It is a care-educational technology with written and visual content based on scientific evidence and articulated with the knowledge of families, organized in a decision tree to guide the FNS of children under six months who are not breastfed. This articulation of knowledge was made possible by the involvement of a team of researchers, specialists in the subject, designers and linguists in the development of the written and visual content in a CT project. The use of this website can be applied in the local context in which the research was carried out or in similar contexts, including the possibility of cultural adaptations of language and illustrations when necessary.

The development of the website met the commitment to guarantee free access, made possible by prototyping the decision tree on an institutional platform, using the resources available at the University and also giving visibility to the authorship linked to the Public University. The production shared with PET-CC allowed undergraduate and postgraduate courses to be linked in an inter-sectoral and interdisciplinary way, through the teaching, research and extension tripod. This link points to the strength of the Public University in producing innovation and training human resources.
