# Supplementary material for: Website for families of non-breastfed children: development and validation of content and interface
Source: Rev Bras Enferm. 2024 Jun 17;77(3):e20230490. doi: 10.1590/0034-7167-2023-0490 (PMC11185075; doi:10.1590/0034-7167-2023-0490)
Supplement: Translation

# Translation: *Website* para família de crianças não amamentadas: desenvolvimento e validação de conteúdo e de interface

**DOI:** 10.1590/0034-7167-2023-0490pt

**Authors:** Marília Alessandra Bick, Cristiane Cardoso de Paula

**Affiliations:** I Universidade Federal de Santa Maria. Santa Maria, Rio Grande do Sul, Brasil

**Keywords:** Segurança Alimentar, Saúde do Lactente, Família, Ciência Translacional Biomédica, Tecnologia Educacional

## Abstract

**Objetivos:**

desenvolver e validar o conteúdo e a interface de *website* orientativo para apoiar a família na promoção de Segurança Alimentar e Nutricional de crianças menores de seis meses não amamentadas.

**Métodos:**

estudo metodológico, de Tradução do Conhecimento, em duas etapas de criação: 1) conteúdo e validação no critério precisão em painel de especialistas; 2) interface e validação nos critérios conteúdo, linguagem, ilustrações, layout, motivação, cultura e aplicabilidade.

**Resultados:**

o *website* “Via láctea” está disponível em livre acesso: https://www.ufsm.br/pet/ciencia-da-computacao/alimentacao-lactea. O conteúdo foi estruturado em árvore de decisão composta por tipo de leite: fórmula láctea, leite de vaca integral e em pó; e utensílios: mamadeira, copinho e colher dosadora. Constam 46 ilustrações para elucidar conteúdo, facilitar compreensão e engajar população-alvo. O Índice de Validade do Conteúdo foi de 0,91.

**Conclusões:**

o *website* é uma tecnologia validada com conteúdo escrito e imagético baseado em evidências e traduzido para uso com famílias.

## INTRODUÇÃO

São poucas as condições em que se recomenda à mulher não amamentar, como mães infectadas pelo HIV, HTLV 1 e 2 ou em uso de medicamento incompatível com a amamentação, a exemplo de tratamento de câncer. Mães usuárias regulares de álcool ou drogas ilícitas não devem amamentar enquanto estiverem fazendo uso destas substâncias. Em casos de mães com distúrbios de consciência ou comportamento grave, o caso deve ser avaliado separadamente. Em quaisquer dessas situações, a alimentação da criança não amamentada precisa necessariamente ser aceitável, viável, acessível, sustentável e segura^(1,2)^. O conceito de segurança alimentar e nutricional (SAN) incorpora, na dimensão alimentar, aspectos relacionados com disponibilidade, produção, comercialização e acesso aos alimentos; e, na dimensão nutricional, aspectos relacionados à seleção, preparo e consumo dos alimentos, sua relação com a saúde e utilização dos nutrientes^(3)^.

Por isso, torna-se imprescindível que a família disponha de água potável, saneamento básico no domicílio e condições financeiras para prover a fórmula láctea infantil e os alimentos em quantidade suficiente para o crescimento adequado da criança. Demanda, também, conhecimentos e habilidades para prepará-los em quantidade e qualidade adequada e oferecê-los de maneira oportuna à idade. Requer acesso periódico aos serviços de saúde para acompanhamento do crescimento e desenvolvimento infantil^(4,5)^.

A exposição dessas crianças à nutrição inadequada em razão da ausência do aleitamento materno amplia a predisposição à morbidade e mortalidade infantil^(6,8)^. Por isso, elas precisam ter garantido o direito a uma alimentação adequada e saudável, e, para que isso seja possível, os familiares precisam receber orientações e informações baseadas em evidências científicas, em tempo oportuno, de forma acessível e sem conflito de interesse, para que consigam adotar práticas seguras de alimentação^(5,9)^.

Entretanto, esses familiares comumente recebem apoio e orientação insuficientes. Por essa razão, a principal fonte de informação das famílias é a própria lata da fórmula infantil, as quais especificam o modo de preparo com uso de mamadeira e diluição da fórmula de acordo com a idade do lactente. As mães, principais responsáveis pela alimentação da criança, demonstram insatisfação com a quantidade e variedade de informações disponíveis para auxiliar na alimentação com fórmula infantil^(5)^.

Os profissionais de saúde são fundamentais para apoiar as famílias de crianças menores de seis meses não amamentadas, informando sobre os tipos de leite e fórmula infantis disponíveis, os tipos de utensílios disponíveis para ofertar o leite, as práticas de higienização dos utensílios, a forma de preparo, quantidades e cuidados necessários para garantir que a criança receba os nutrientes necessários para um adequado crescimento e desenvolvimento^(5)^. A enfermeira e o pediatra nos serviços de atenção primária à saúde devem orientar a família dessas crianças conforme as recomendações sobre alimentação de crianças nos primeiros anos de vida, que sustentam os profissionais no desenvolvimento de ações individuais e coletivas de educação alimentar e nutricional no Sistema Único de Saúde^(2)^. Os agentes comunitários de saúde deverão acompanhar as demandas dessas famílias e das crianças nas visitas domiciliares.

A prática alimentar cotidiana dos lactentes se modifica conforme a idade e nem sempre os familiares conseguem ter acesso aos profissionais de saúde para esclarecer suas dúvidas do dia a dia, sobretudo ao se considerar o fato de o cronograma de consultas de puericultura ser mensal nos primeiros seis meses de vida. Tecnologias educativas se mostram como uma alternativa que pode auxiliar no fortalecimento de orientações, mas há lacuna dessas ferramentas atualizadas e baseadas em evidências científicas acerca da alimentação de crianças não amamentadas. Por isso, muitas vezes, os pais e/ou demais pessoas envolvidas com as práticas alimentares cotidianas dos lactentes recebem informações conflitantes e de fontes inadequadas^(4,9)^.

Em particular, as tecnologias digitais atendem as crescentes necessidades de informação da população, que tentam gerir a própria saúde utilizando recursos online. Diversas ferramentas digitais são capazes de possibilitar uma interação entre a população e o sistema de saúde, constituindo um aliado válido. Assim, a literatura descreve que o uso da web é uma estratégia de sucesso para acesso a orientações de saúde^(10)^.

Entretanto, há necessidade oferecer uma fonte confiável de informações baseadas em evidências científicas e de traduzir as orientações para promover a nutrição e o desenvolvimento infantil saudável. Considerando a ampla disponibilidade de evidências científicas existentes e a dificuldade de acesso da população a essas informações, apresentá-las em linguagem acessível e ajustada às orientações dos profissionais de saúde permite qualificar os cuidados de saúde e aproximar a pesquisa da prática clínica.

## OBJETIVOS

Desenvolver e validar o conteúdo e a interface de um *website* orientativo para apoiar as famílias na promoção de Segurança Alimentar e Nutricional de crianças menores de seis meses não amamentadas.

## MÉTODOS

### Aspectos éticos

Esta pesquisa seguiu as Resoluções nº 466/2012, 510/2016 e 674/ 2022, com projeto matricial aprovado pelo Comitê de Ética em Pesquisa. A privacidade e o sigilo das informações pessoais foram garantidos pelo Termo de Confidencialidade dos dados por meio do envio de mensagens e e-mails de forma individual, aplicação o termo de consentimento livre e esclarecido de maneira eletrônica, sendo o questionário possível de ser respondido mediante o aceite eletrônico do participante, com esclarecimento da possibilidade de abster da pesquisa a qualquer momento sem nenhum prejuízo moral ou legal, e armazenamento do banco de dados de forma segura que impossibilitem a identificação do participante.

### Desenho, período e local do estudo

O desenho desta pesquisa é parte de projeto matricial de tradução do conhecimento (TC)^(11)^ intitulado “Tradução de conhecimentos na produção de tecnologias educativas promotoras de cuidado de crianças em risco de exposição ao HIV” (CAPFAM III), desenvolvido pelo Grupo de Pesquisa Cuidado à Saúde das Pessoas, Famílias e Sociedade (GP-PEFAS). No subprojeto que originou este artigo, foi desenvolvido o ciclo de criação do Modelo de TC (Figura 1).

**Figura 1:** Aplicação das fases do ciclo de criação no Projeto de Tradução do Conhecimento, Santa Maria, Rio Grande do Sul, Brasil, 2022

*RI - Revisão Integrativa; INSAN - Insegurança Alimentar e Nutricional; SAN - Segurança Alimentar e Nutricional; RSQ - Revisão Sistemática Qualitativa; PET-CC – Programa de Educação Tutorial do Curso de Graduação em Ciências da Computação; PP - Projeto de Pesquisa; CAPFAM - Avaliação da capacidade para cuidar de crianças expostas ao HIV.*

*Fonte: Graham, Logan, Hanson, Strauss, Tetroe, Caswell, Robinson^(12)^ (traduzido por Vieira, Gastaldo, Harrison^(13)^).*

O período de criação desta tecnologia se deu em um total de 40 meses e contemplou: 1) etapa de criação do conteúdo (desenvolvimento do texto, validação e desenvolvimento das imagens) e 2) etapa de criação da interface (desenvolvimento do website e validação) (Figura 2).

**Figura 2:** Fluxograma metodológico do Projeto de Tradução do Conhecimento, Santa Maria, Rio Grande do Sul, Brasil, 2022

O desenvolvimento do conteúdo (fase de síntese do conhecimento) iniciou em dezembro de 2019, após a atualização do Guia alimentar para crianças brasileiras menores de 2 anos^(2)^. O conteúdo textual e imagético foi desenvolvido em 24 meses e a fase foi concluída em dezembro 2021 com a validação do conteúdo em painel de especialistas. O desenvolvimento da interface (fase de produto do conhecimento) iniciou em abril 2022 e teve duração de 12 meses, contemplando validação da interface por especialistas em novembro de 2022. Em maio de 2023 o *website* obteve número de registro e foi divulgado em evento de lançamento.

O local de estudo é uma instituição de ensino superior pública e federal brasileira, do interior do estado, que tem a missão construir e difundir conhecimento, comprometida com a formação de pessoas capazes de inovar e de contribuir com o desenvolvimento da sociedade, de modo sustentável. Tal missão, coerente com o Plano de Desenvolvimento Institucional, que inclui o tópico inovação e transferência de tecnologias nas diretrizes das políticas de ensino, pesquisa e extensão, potencializou o apoio institucional no desenvolvimento dessa tecnologia e a articulação intersetorial e multidisciplinar a para composição da equipe do projeto de TC.

### População ou amostra; critérios de inclusão e exclusão

Para a validação do conteúdo, foi realizado um painel de especialistas, os quais foram selecionados por meio da técnica de Bola de Neve. Trata-se de amostra não probabilística, sendo que a escolha dessa técnica se deu devido à necessidade de avaliação por profissionais com conhecimento e experiência na temática de alimentação e na população com faixa etária menor de seis meses.

A composição do painel de especialistas com representantes de profissionais de saúde, docentes, gestores municipais, estaduais e federais com experiência no tema foi um desafio que se trilhou pelo caminho do Modelo de TC, que prevê o engajamento dos tomadores de decisão. O desafio foi alcançado por meio dos contatos estabelecidos durante o desenvolvimento dos projetos que compõem o núcleo de segurança alimentar e nutricional (NUSAN) do GP, inclusive das inúmeras oportunidades em que o projeto matricial CAPFAM foi apresentado em eventos, inclusive premiado, o que lhe conferiu visibilidade nacional e internacional.

Inicialmente, o convite foi enviado para coordenação técnica geral do Guia^(2)^, Coordenação-Geral de Vigilância do HIV do Ministério da Saúde, Coordenadoria Regional de Saúde e Secretaria de Saúde do Município. O convite foi enviado por e-mail e, ao recebê-lo, o profissional poderia indicar outro(s) especialista(s) para compor o painel, aos quais também foi enviado o convite. Foram convidados 25 profissionais de saúde, de diferentes locais e instituições do país. Participaram do painel 13 especialistas, os quais eram vinculados às instituições: Coordenação Geral de Alimentação e Nutrição (CGAN) do Ministério da Saúde; Rede Internacional em Defesa do Direito de Amamentar (IBFAN); Fundo das Nações Unidas para a Infância (UNICEF); Conselho Nacional de Segurança Alimentar e Nutricional (CONSEA); Organização Pan-Americana da Saúde (OPAS); Núcleo de resposta à TV do HIV do Ministério da Saúde; Política de Alimentação e Nutrição do estado do Rio Grande do Sul; 4ª Coordenadoria Regional de Saúde; Secretaria Municipal de Saúde; Política Municipal HIV/Aids; e Pediatria do Hospital Universitário de Santa Maria.

Para a validação da interface do *website*, o recrutamento dos participantes se deu novamente pela técnica Bola de Neve. Inicialmente, o convite foi enviado aos 13 especialistas que participaram da validação de conteúdo, sendo que os que aceitaram participar da etapa de validação da interface indicaram outros especialistas no tema. No total, foram enviados 77 convites, dos quais 28 especialistas aceitaram participar.

### Protocolo do estudo

Este estudo foi desenvolvido em duas etapas: 1) criação do conteúdo (texto, linguagem, ilustrações e identidade visual) baseado em evidências científicas; e 2) desenvolvimento da interface (layout e operacionalidade), ambas as fases validadas por especialistas.

A primeira etapa iniciou com uma oficina na instituição de ensino superior, com representantes de familiares de crianças expostas ao HIV, que não recebem leite materno. Os familiares receberam o convite para participar por meio de um projeto de extensão desenvolvido pelo grupo de pesquisa, no serviço de saúde especializado no Hospital Universitário, e a participação foi voluntária. Nessa oficina, apresentou-se a estruturação do conteúdo no formato de perguntas e orientações. A intenção foi verificar se a proposta do website atendia às necessidades da população-alvo e se estava coerente com suas demandas e o contexto local. Os participantes falaram sobre a experiência de alimentar as crianças, suas dificuldades e a realidade de seu dia a dia, o que subsidiou a elaboração do conteúdo textual e imagético.

Para o desenvolvimento do conteúdo textual, selecionou-se os pontos-chave das evidências científicas e do Guia. Posteriormente, fez-se a estruturação da árvore de decisão em que os pontos-chave foram agrupados por tipo de leite. O conteúdo da árvore foi composto por perguntas, com opção de respostas dicotômicas (sim e não), sendo que, na opção, não foi elaborado o conteúdo orientativo para promoção de SAN. Para elaboração da escrita das perguntas, considerou-se as experiências das famílias para o preparo e oferta da alimentação láctea, acessadas na oficina com o público-alvo.

Posteriormente, foi realizado o painel de especialistas para revisão linguística. Essa etapa foi feita de maneira virtual, por meio de duas reuniões com uma especialista da área de Letras e outra especialista da área de Publicidade e Propaganda. Após a revisão linguística, procedeu-se com o painel, contando com a colaboração de duas especialistas do Grupo de Monitoramento da Norma Brasileira de Comercialização de Alimentos para Lactentes e Crianças de Primeira Infância, Bicos, Chupetas e Mamadeiras (NBCAL). Nesse painel, fez-se ajustes no texto e nas imagens no material, de modo a atender às recomendações de normalização.

A validação do conteúdo textual aconteceu no formato de painel de especialistas em alimentação infantil. O convite foi enviado por e-mail, o qual continha a apresentação dos objetivos do projeto de pesquisa, a indicação das possíveis datas para a realização do painel e o link de uma agenda eletrônica (*Doodle*) para a seleção do melhor turno e data de realização. Após a confirmação de aceite, foi enviado o arquivo para apreciação prévia.

No painel de discussão do conteúdo textual, desenvolveu-se a revisão por especialistas para evidências de validade de conteúdo, que atentou ao critério de precisão^(14)^ das perguntas e orientações. Os participantes enviaram as sugestões de ajustes que foram compiladas (e destacadas em letra vermelha) em um arquivo único pela mediadora do painel. O arquivo foi compartilhado na tela da reunião, realizada dentro de um período de dois dias, totalizando sete horas. A mediadora procedeu à leitura do conteúdo de cada pergunta. Durante o processo, eles tiveram a oportunidade de sugerir exclusão, acréscimo e alterações no conteúdo textual e imagético. Quando havia indicação de alteração por algum especialista, caso o ajuste no texto procedesse e após o consenso no painel, dava-se seguimento para a validação da pergunta seguinte da árvore de decisão.

Contratou-se um ilustrador com o qual foram realizadas reuniões virtuais. A comunicação foi mantida por aplicativo de mensagens para otimizar a comunicação. Foi feito um roteiro de acordo com o conteúdo validado das perguntas e orientações, detalhando-se as características necessárias para cada imagem. Inicialmente, o ilustrador apresentou um mapa mental com palavras-chave que o auxiliaram na construção das ilustrações e dos esboços das imagens, dos tipos de leite e dos utensílios para alimentar a criança. Após, foram criados os personagens. Na sequência, apresentou-se as imagens e a equipe da pesquisa solicitou os ajustes. Utilizou-se o programa Adobe Photoshop através de uma mesa digitalizadora.

A equipe de pesquisa e o ilustrador trabalharam conjuntamente na escolha do nome do *website*, dessa forma, foram realizadas pesquisas de termos que remetessem ao conteúdo, sem prejuízo ao cumprimento da NBCAL. Assim, o ilustrador apresentou a identidade visual da tecnologia. Então, foram feitos testes para a escolha das cores principais da paleta. Também fez-se um levantamento de cores para a representação de cor de pele, sendo o mais abrangente possível, visando à diversidade. Com a definição da paleta de cores, a identidade visual e as ilustrações foram finalizadas com adição de cores, luzes e sombras.

A segunda etapa iniciou a partir da parceria com uma equipe de cientistas da computação, vinculados ao Programa de Educação Tutorial da Ciência da Computação (PET-CC). Os encontros virtuais possibilitaram a apresentação da proposta, esclarecimento de dúvidas e demandas de ajustes. A comunicação foi mantida por e-mail e aplicativo de mensagens. Elaborou-se o roteiro da árvore de decisão com as perguntas, o conteúdo da resposta ‘sim’ e a orientação da resposta ‘não’, com suas respectivas imagens. Para o desenvolvimento da interface, a equipe do PET-CC desenvolveu um piloto do *website* com conteúdo textual, o qual foi acessado pela equipe de pesquisa que elaborou um relatório dos ajustes necessários. Após esse processo, incluiu-se a identidade visual e as ilustrações. Para a criação e manutenção do *website,* utilizou-se a plataforma *WordPress*. Quanto à edição detalhada e personalizada das páginas, foi utilizada a ferramenta *Elementor*. A escolha por esse *software* deu-se pela necessidade de hospedagem no site institucional da Universidade, que utiliza a plataforma em questão.

A equipe da pesquisa procedeu o teste de rastreamento de problemas no *website*, percorrendo a árvore de decisão e acessando os *links*, gerando um segundo relatório de ajustes necessários. Por fim, a equipe de pesquisa procedeu o primeiro teste de usabilidade, de forma que o *website* foi acessado via celular, tablet (Android e IOS) e computador, buscando identificar necessidades de ajustes de fonte, cores e correções do sistema. As inconformidades identificadas foram comunicadas. Algumas funcionalidades desejadas pela equipe não foram possíveis de realizar por limitação da plataforma institucional, como a impossibilidade de leitura da página completa, quando acessada por meio de *smartphone* com sistema operacional *Android*.

A validação da interface aconteceu no formato de painel de especialistas. O convite foi enviado por um *e-mail*, apresentando o objetivo da pesquisa, o método utilizado para a validação e o *link* de acesso ao *Google Forms*. Para assegurar a participação de um maior número de especialistas, a cada cinco dias foram enviados *e-mails* de lembrete sobre a pesquisa para os participantes que ainda não haviam respondido. Também utilizou-se o contato via aplicativo de *WhatsApp*. A validação foi realizada por meio de um formulário *online*, utilizando a plataforma *Google Forms*, vinculado a um *e-mail* institucional da pesquisadora.

A primeira parte do formulário consistiu na caracterização dos especialistas, com sete perguntas. Na sequência, o participante foi convidado a acessar e percorrer o *website* e, posteriormente, responder ao formulário de validação, já aplicado em tecnologia educativa para famílias^(15)^. Os critérios de validação foram: a) conteúdo - com cinco itens; b) linguagem - com três itens; c) ilustrações - com cinco itens; d) layout - com sete itens; e) motivação - com três itens; f) cultura - com dois itens; g) aplicabilidade - com um item. Para cada questão, empregou-se uma escala do tipo *Likert*, com as opções: 1 -Inadequado; 2 - Parcialmente adequado; 3 - Adequado; 4 - Totalmente adequado. Ao final de cada bloco de perguntas, disponibilizou-se um espaço para que os especialistas realizassem apontamentos de sugestões e opiniões para aprimoramento da tecnologia.

### Análise dos resultados e estatística

As respostas foram exportadas da plataforma *Google Forms,* no formato de arquivo do *software Microsoft Excel*, para o programa de análise SPSS v. 20.0. As variáveis categóricas foram descritas por frequências e percentuais. A normalidade das variáveis quantitativas foi avaliada através do teste de Shapiro Wilk. As variáveis quantitativas com distribuição normal foram descritas pela média, o desvio padrão (idade) e aquelas com distribuição assimétrica foram descritas pela mediana e pelo intervalo interquartil (tempos de atuação e formação).

O Índice de Validade do Conteúdo (IVC) foi calculado para cada um dos itens, das categorias e global. Para calcular o IVC das categorias – conteúdo, linguagem, ilustração, layout, cultura e aplicabilidade – foram utilizadas as médias. O IVC mede a porcentagem de concordância dos especialistas sobre determinados aspectos do instrumento e de seus itens. É possível calcular o IVC para cada item e o total da escala. Para o cálculo, é necessário que a escala tenha quatro pontos (1 - Inadequado; 2 - Parcialmente adequado; 3 - Adequado; 4 - Totalmente adequado), são somadas as respostas 3 e 4 e o resultado dessa soma é dividido pelo número total de respostas. Um IVC aceitável para cada um dos itens deve ser de no mínimo 0.78, já para a escala global, o índice aceitável é de 0.80. Valores menores indicam necessidade de revisão ou rejeição dos itens^(16)^.

Foi calculado o *alpha* de *Cronbach* do instrumento. O valor mínimo aceitável para a confiabilidade é de 0,70^(17)^. *O alpha* de *Cronbach* foi alto, equivalente a 0,95. Esse resultado indica que, apesar de não ter sido construído para este tema e nem para esta população específica, o instrumento utilizado foi adequado para a coleta de dados, apresentando consistência interna dos itens.

## RESULTADOS

A identidade visual do *website* é “Via Láctea: o caminho para segurança alimentar e nutricional para crianças menores de seis meses não amamentadas”. A escolha sustentou-se na identificação de que a Galáxia Via Láctea foi assim batizada pelos Gregos por causa do seu aspecto esbranquiçado e era vista como um caminho de leite. Portanto, o nome “Via Láctea” remete tanto ao caminho, seja aquele percorrido pela família para alimentar a criança que não é amamentada, seja a via de oferta dessa alimentação. E o termo láctea é definido pelo dicionário como “que contém leite, composto por leite”.

As cores verde e rosa foram elencadas como as principais, considerando que, por serem complementares, elas possuem contraste e destacam os elementos nas imagens. Buscou-se cores semelhantes ao rosa e, para ampliar as possibilidades, foram incluídos o marrom e o amarelo. Os tons variam entre vibrantes, cores saturadas e tons pastéis.

A tela inicial do *website* contém uma breve apresentação do objetivo da tecnologia, do público-alvo, da justificativa acerca da necessidade de orientar familiares sobre SAN para crianças menores de seis meses não amamentadas e o que significa SAN. Há um *link* que redireciona os usuários ao Guia alimentar para crianças brasileiras menores de 2 anos, principal fonte de conteúdo desta tecnologia. Além disso, a tela inicial também reforça a necessidade de a família procurar um profissional de saúde, disponibilizando *links* para acesso à Caderneta de Saúde da Criança.

Na segunda tela, está a árvore de decisão, em que o familiar pode fazer a escolha do caminho a seguir no *website*, conforme a sua necessidade de orientação. O conteúdo textual foi estruturado em perguntas e respostas, com escolhas por tipo de leite: fórmula láctea (com 4 perguntas), leite de vaca integral e em pó (com 6 perguntas cada); e utensílios: mamadeira, copinho e colher dosadora (com 10 perguntas cada), como se pode ver na Figura 3.

**Figura 3:** Identidade visual e árvore de decisão do *website*, Santa Maria, Rio Grande do Sul, Brasil, 2022

Constam 46 ilustrações para elucidar conteúdo, facilitar compreensão e engajar população-alvo. Foram criados ícones para identificar os três tipos de leite e os três tipos de utensílios utilizados para alimentar a criança. Os personagens são: uma criança menor de seis meses, uma profissional de saúde, uma mãe, um pai e um irmão na faixa etária escolar. Também foram criadas imagens de materiais que as famílias utilizam em seu dia a dia para preparo do leite e para higiene dos utensílios. As ilustrações foram articuladas ao conteúdo textual, como pode ser visualizada no exemplo de algumas telas orientativas na Figura 4.

**Figura 4:** Mosaico de telas orientativas do *website* referentes a opção de utensílio copinho para o leite de vaca integral, Santa Maria, Rio Grande do Sul, Brasil, 2022

No rodapé de cada tela, há a possibilidade de clicar na opção sumário que redireciona o usuário para a tela da árvore de decisão e permite que seja realizada novamente a seleção do tipo de leite e utensílio utilizado para alimentar a criança. Assim, o percurso pode ser realizado quantas vezes for necessário.

A tela final do *website* consta dos créditos de autoria, ilustração, programação do *website*, revisão textual, adequação a NBCAL e especialistas do painel de validação. Além disso, há o selo de registro de obra, informação de premiação do projeto de inovação e das agências de fomento.

O *website* obteve Índice de Validade do Conteúdo de 0,91. Os critérios conteúdo, linguagem, ilustração, *layout*, motivação, cultura e aplicabilidade obtiveram o IVC dentro do parâmetro previamente estabelecido (Tabela 1). Apesar de o *website* apresentar um IVC global adequado, as alterações propostas pelos especialistas nas imagens e no texto foram atendidas para a versão final.

**Tabela 1:** Distribuição do Índice de Validação de Conteúdo no painel de especialistas, Santa Maria, Rio Grande do Sul, Brasil, 2022

CRITÉRIOS/Itens	IVC
CONTEÚDO	0,85
A1. O conteúdo está correto cientificamente	0,86
A2. O conteúdo está apropriado ao público-alvo	0,75
A3. O conteúdo é suficiente para atender às necessidades do público-alvo	0,82
A4. A sequência do texto é lógica	0,89
A5. A apresentação do conteúdo favorece a aprendizagem da temática	0,93
LINGUAGEM	0,89
B1. O estilo da redação é compatível como público-alvo	0,96
B2. A escrita utilizada é atrativa	0,89
B3. A linguagem do texto é clara e objetiva	0,82
ILUSTRAÇÃO	0,93
C1. As ilustrações são pertinentes e elucidam o conteúdo	0,86
C2. As ilustrações são claras e transmitem facilidade de compreensão	0,89
C3. As ilustrações possuem qualidade gráfica	0,96
C4. A quantidade de ilustrações está adequada para o conteúdo do material	0,93
C5. A presença de cada uma das figuras é relevante	1,00
LAYOUT	0,94
D1. O tipo de letra utilizado facilita a leitura	0,96
D2. As cores aplicadas ao texto são pertinentes e facilitadoras para a leitura	1,00
D3. A composição visual está atrativa e bem organizada	0,93
D4. O formato do material educativo está adequado	0,90
D5. A disposição do texto está adequada	0,89
D6. O tamanho das letras dos títulos, subtítulos e texto é adequado	0,96
D7. O tamanho da tecnologia está adequado	0,93
MOTIVAÇÃO	0,94
E1. O conteúdo está motivador e incentiva prosseguir a leitura	0,93
E2. O conteúdo despertou o interesse do leitor	0,96
E3. O conteúdo poderá atender às dúvidas, esclarecer e educar o familiar	0,93
CULTURA	0,87
F1. O texto está compatível com o público, atendendo aos diferentes perfis	0,82
F2. A tecnologia está indicada para uso como recurso de apoio à família	0,93
APLICABILIDADE	0,93
G1. A tecnologia possui aplicabilidade na prática	0,93
Total	0,91

*IVC – Índice de Validação de Conteúdo.*

Responderam à validação 28 especialistas, dos quais 27 (96,4%) são do sexo feminino e 1 (3,6%) do sexo masculino. A idade dos participantes apresentou uma média de 43,4 anos. Em relação à área de formação 15 (53,6%) eram da Enfermagem, 9 (32,1%) da Nutrição e 4 (14,3%) da medicina. Quanto à área de atuação, 23 (82,1%) atuam no ensino, 20 (71,4%) na pesquisa e 11 (39,3%) na assistência. Ressalta-se que nesse item, os especialistas tiveram a possibilidade de assinalar mais de uma opção. A mediana do tempo de formação dos participantes foi de 16 anos, variando de no mínimo 10 e no máximo 24 anos. Quanto à mediana do tempo da última titulação dos participantes foi de 7,5 anos, variando de no mínimo 4,0 anos e no máximo 10,7 anos.

Com o conteúdo textual, imagético e a interface validados, o *website* obteve número de registro de obra 7121883. Está disponível em domínio institucional de livre acesso: https://www.ufsm.br/pet/ciencia-da-computacao/alimentacao-lactea

## DISCUSSÃO

O *website* foi validado para uso com famílias na promoção de SAN de crianças menores de seis meses não amamentadas. Parâmetros semelhantes de IVC global foram encontrados na validação de outras tecnologias educativas brasileiras, variando entre 0,84-0,99 que apontam o alinhamento do resultado obtido no painel de especialistas com os índices de estudos metodológicos anteriores de tecnologias educativas no tema^(18)^ e na população-alvo^(19)^.

A validação do critério conteúdo indica que os especialistas consideraram o texto está coerente com as evidências científicas atuais, com sequência lógica adequada, e que favorece a aprendizagem na temática. Em outras pesquisas metodológicas realizadas no país, os especialistas também consideraram o conteúdo das tecnologias como adequadas^(20,21)^. O item que obteve escore menor que o ponto de corte foi a adequação do conteúdo ao público-alvo, sem inviabilizar a validação do critério. A principal sugestão realizada pelos especialistas nesse item refere-se à adequação do texto contendo termos técnicos. O resultado indica a pertinência da próxima etapa do projeto matricial, qual seja: validação da tecnologia com o público-alvo^(22)^, para que sejam contemplados os ajustes ao contexto local, conforme preconizado no Modelo de TC^(11)^.

A validação do critério de linguagem indicou que o estilo de redação está compatível com o público-alvo, que a escrita é atrativa, clara e objetiva. Em uma tecnologia educativa, a linguagem é fundamental para que a mensagem seja compreendida corretamente e seja aplicável no dia a dia dos indivíduos. Por essa razão, a linguagem deve ser adequada ao nível educacional e cultural da população a que se destina, rompendo a barreira entre conhecimento científico e conhecimento tácito^(21,23)^. Cabe ressaltar que a utilização de uma tecnologia educativa depende também de uma linguagem atrativa, que chame a atenção do usuário do conhecimento traduzido na ferramenta. Caso contrário, o indivíduo pode buscar informações e orientações em outras fontes, as quais nem sempre são apropriadas às necessidades e sustentadas em evidências científicas^(21,22,24)^. O acesso a esse tipo de informação pode ampliar o risco de insegurança alimentar de crianças não amamentadas. A linguagem deve ser objetiva para assegurar que o familiar disponha de tempo para consultar as informações e esclarecer dúvidas, atendendo à praticidade que um cuidador de criança com menos de seis meses precisa^(4,5)^.

A validação do critério ilustração apontou que as ilustrações do *website* foram consideradas de qualidade, pertinentes, claras, elucidativas ao conteúdo e como facilitadoras da compreensão do texto. O uso de ilustrações em tecnologias educativas desperta o interesse do usuário e auxilia na compreensão do conteúdo. Resultados semelhantes foram encontrados no desenvolvimento de recursos educativos para o aleitamento materno^(18)^ e para prevenção de excesso de peso^(21)^.

A validação do critério *layout* indica que os especialistas consideraram que o *website* apresenta uma composição visual atrativa, com letras e cores que facilitam a leitura do texto, com disposição do tamanho de letras e tamanho de textos adequados. Dois tipos de fontes são utilizados no *website:* a *Rawline* e a *Sans Serif*, o fundo da página é branco e alguns detalhes encontrados na página estão relacionados à hospedagem do *website* em um domínio institucional da UFSM. Outras pesquisas brasileiras que construíram e validaram tecnologias educativas do tipo *m-Health*, nos mais variados temas, reforçam a importância de apresentar um *layout* atrativo e relativamente simples que permita ao usuário o acesso independentemente da velocidade de sua rede móvel ou espaço de armazenamento no dispositivo^(22,25)^.

Para o critério motivação, os especialistas consideram que o conteúdo incentiva a leitura, desperta o interesse e tem potencial de esclarecer dúvidas de familiares. Resultados semelhantes foram encontrados em estudos brasileiros que validaram tecnologias educacionais para familiares de crianças. Na validação do dispositivo móvel sobre aleitamento materno para a população de familiares de recém-nascidos, o critério motivação obteve IVC 0,95^(22)^. A validação de um pacote de mensagens de texto e imagens para promoção do aleitamento materno obteve IVC 0,86 na motivação^(21)^.

Em relação ao critério cultura, os especialistas validaram o texto como compatível à população, indicando seu uso como recurso para apoiar a família na promoção da SAN de crianças menores de seis meses não amamentadas. Estudos de validação de tecnologias educativas também apresentaram escores de IVC acima de 0,80 para o critério cultura, o que indica a adequação para utilização com a população brasileira^(22,26)^. Reitera-se que os especialistas que participaram da validação do *website* Via Láctea, são de diversas regiões do país. A validação do último critério, aplicabilidade na prática, aponta o potencial de uso do website. Os especialistas indicaram a possibilidade do uso da tecnologia na prática das consultas de enfermagem^(23)^.

A validação, por especialistas, das tecnologias baseadas em evidências científicas potencializa a sua utilização, uma vez que são eles os profissionais que atuam na assistência à saúde dos indivíduos e que podem efetivamente mediar o uso do recurso educacional^(21,23)^. Assim, para tornar a tecnologia mais completa, adequada à população que se destina e com maior rigor científico para uso na educação em saúde, considerou-se as sugestões indicadas pelos especialistas, o que também foi identificado em outras pesquisas^(20,23)^. As sugestões contribuíram para aprimorar a tecnologia em termos de conteúdo textual e imagético tanto em coerência com as evidências científicas quanto com a tradução do conhecimento à população-alvo de familiares de crianças menores de seis meses não amamentadas e ao contexto local do Sistema Único de Saúde brasileiro.

A qualidade de uma tecnologia cuidativo-educacional^(27)^ transcorre da utilização de evidências científicas e a validação perpassa por especialistas para que estejam em conformidade com a prática^(28)^. Outros estudos brasileiros que desenvolveram e validaram tecnologias educativas também utilizaram o IVC, indicando a convergência do método utilizado para qualificar a produção desses recursos educacionais^(20,24,29)^. Para incorporar as tecnologias aos diferentes cenários, é imprescindível que elas sejam validadas e que o pesquisador se comprometa em mantê-las em permanente avaliação e monitoramento^(12,13)^. O desenvolvimento de tecnologias realizado por equipes multiprofissionais amplia a gama de conhecimentos científicos e práticos para aprimorar os produtos^(20,24,30)^. A inserção de profissionais que atuam tanto na área do ensino, quanto na área da pesquisa e/ou da assistência pode balizar a utilização da tecnologia como ferramenta de promoção da saúde^(20,21)^.

Considerando o modelo de TC para atender o compromisso de disseminação da ferramenta do conhecimento, o lançamento da tecnologia educativa foi desenvolvido por meio de uma ação de extensão do GP denominada Seminário de Integração da Pesquisa com a Prática (SIPP), desenvolvida no encerramento de projetos matriciais para o compartilhamento dos resultados com a comunidade. Na edição, o SIPP foi feito no formato de aula aberta intitulada “Segurança alimentar de crianças menores de seis meses”, cuja divulgação foi feita pelas redes sociais do GP, do centro de ciências da saúde e da pró-reitoria de extensão da Universidade. Contou com a audiência de estudantes de graduação e residência da área da saúde, profissionais da atenção primária e hospitalar, docentes e gestores. E a cobertura do evento foi feita pela TV Campus e noticiada em https://www.youtube.com/watch?v=73FRgE0RPPU.

Conforme o Modelo de TCA preconiza o engajamento dos *stakeholders* para a manutenção do uso do produto do conhecimento, no SIPP retomou-se a participação de gestores dos setores do ensino, da pesquisa e da extensão da Universidade, da área materno infantil do Hospital Universitário, da Secretaria de Saúde do Município e da 4ª Coordenadoria Regional de Saúde (CRS). No SIPP, foi feita a entrega simbólica do *website* aos gestores por meio de card com QR CODE de acesso (Figura 5). Também foi produzido um vídeo com a equipe de produção do *website* e com os gestores que o apoiaram como ferramenta que poderá ser utilizada, inclusive, de modo didático, como ilustração do processo de engajamento para TC. O vídeo pode ser acessado em https://youtu.be/d9VJfRG1JkU?si=kAZavT9wR_Y5SyZp.

**Figura 5:** Arte do kit de divulgação do *website*, Santa Maria, Rio Grande do Sul, Brasil, 2022

Essa mesma arte é impressa no formato de cartaz, disponibilizado para divulgação nos serviços. Para cada município da 4ª CRS foi enviado um kit de disseminação do *website*. O quantitativo de *cards* e de cartazes foi calculado segundo o número de unidades de saúde, acompanhados de ofício do GP, no qual consta também o *link* do vídeo curto de divulgação do *website*, disponível em https://www.instagram.com/reel/CurPY2Xu-Rb/?igsh=NzBmMjdhZWRiYQ. Esses produtos de divulgação são resultado deste projeto de TC e expressam o compromisso de continuidade de disseminação, pois há a demanda de investimento para manutenção do uso dessa tecnologia pelas famílias de crianças menores de seis meses não amamentadas.

### Limitações do estudo

A limitação do presente estudo foi consequência do isolamento social, devido à pandemia da COVID-19, que se estabeleceu como um empecilho para a inserção da população-alvo em todo o processo de desenvolvimento do conteúdo e interface do *website*, conforme a recomendação do Modelo de TC para que haja engajamento da audiência do produto do conhecimento. Portanto, indicamos a necessidade de avaliação dessa tecnologia pelos próprios familiares, etapa que está prevista no cronograma de execução do projeto matricial CAPFAM III.

### Contribuições para a Área

A contribuição é de caráter de inovação do Modelo de TC, com a produção de uma tecnologia para educação alimentar e nutricional segundo as necessidades do grupo vulnerável de crianças menores de seis meses não amamentadas. O *website* poderá ser utilizado pelo familiar no seu cotidiano de prática alimentar da criança. O profissional poderá mediar o uso como uma ferramenta para retomar orientações e esclarecer dúvidas dos familiares. Os profissionais podem ser capacitados para o uso do website, inclusive atualizando sua prática baseada em evidências científicas atuais.

## CONCLUSÕES

O *website* foi validado em conteúdo, linguagem, ilustrações, layout, motivação, cultura e aplicabilidade. É uma tecnologia cuidativo-educacional, com conteúdo escrito e imagético baseado em evidências científicas e articulado com os conhecimentos das famílias, organizado em árvore de decisão para orientar a SAN de crianças menores de seis meses que não são amamentadas. Essa articulação de conhecimentos foi possível pelo engajamento de equipe de pesquisadores, especialistas no tema, técnicos de designer e de linguística na elaboração do conteúdo escrito e imagético em projeto de TC. O uso deste *website* poderá ser aplicado no contexto local em que a pesquisa foi desenvolvida ou ainda em contextos semelhantes, inclusive com a possibilidade de adaptações culturais de linguagem e de ilustrações, quando necessárias.

O desenvolvimento do *website* atendeu o compromisso de garantir o acesso gratuito, possível ao prototipar a árvore de decisão em plataforma institucional, utilizando os recursos disponíveis na Universidade e conferindo, inclusive, visibilidade da autoria vinculada à Universidade Pública. A produção compartilhada com o PET-CC permitiu a articulação entre graduação e pós-graduação, de modo intersetorial e interdisciplinar, por meio do tripé ensino, pesquisa e extensão. Essa articulação aponta a fortaleza da Universidade Pública na produção de inovação e de formação de recursos humanos.
